# Tauroursodeoxycholic Acid (TUDCA) Protects Photoreceptors from Cell Death after Experimental Retinal Detachment

**DOI:** 10.1371/journal.pone.0024245

**Published:** 2011-09-22

**Authors:** Dimosthenis Mantopoulos, Yusuke Murakami, Jason Comander, Aristomenis Thanos, Miin Roh, Joan W. Miller, Demetrios G. Vavvas

**Affiliations:** Retina Service, Angiogenesis Laboratory, Massachusetts Eye and Ear Infirmary, Department of Ophthalmology, Harvard Medical School, Boston, Massachusetts, United States of America; University of Oldenburg, Germany

## Abstract

**Background:**

Detachment of photoreceptors from the underlying retinal pigment epithelium is seen in various retinal disorders such as retinal detachment and age-related macular degeneration and leads to loss of photoreceptors and vision. Pharmacologic inhibition of photoreceptor cell death may prevent this outcome. This study tests whether systemic administration of tauroursodeoxycholic acid (TUDCA) can protect photoreceptors from cell death after experimental retinal detachment in rodents.

**Methodology/Principal Findings:**

Retinal detachment was created in rats by subretinal injection of hyaluronic acid. The animals were treated daily with vehicle or TUDCA (500 mg/kg). TUNEL staining was used to evaluate cell death. Photoreceptor loss was evaluated by measuring the relative thickness of the outer nuclear layer (ONL). Macrophage recruitment, oxidative stress, cytokine levels, and caspase levels were also quantified. Three days after detachment, TUDCA decreased the number of TUNEL-positive cells compared to vehicle (651±68/mm^2^ vs. 1314±68/mm^2^, P = 0.001) and prevented the reduction of ONL thickness ratio (0.84±0.03 vs. 0.65±0.03, P = 0.002). Similar results were obtained after 5 days of retinal detachment. Macrophage recruitment and expression levels of TNF-a and MCP-1 after retinal detachment were not affected by TUDCA treatment, whereas increases in activity of caspases 3 and 9 as well as carbonyl-protein adducts were almost completely inhibited by TUDCA treatment.

**Conclusions/Significance:**

Systemic administration of TUDCA preserved photoreceptors after retinal detachment, and was associated with decreased oxidative stress and caspase activity. TUDCA may be used as a novel therapeutic agent for preventing vision loss in diseases that are characterized by photoreceptor detachment.

## Introduction

Photoreceptor loss occurs acutely after retinal detachment. Although surgery is performed for rhegmatogenous retinal detachment the visual acuity of patients is not always restored after successful reattachment surgery. [1]–[3] In other retinal disorders including age-related macular degeneration and diabetic retinopathy, retinal photoreceptor detachment persists chronically and vision loss progresses for many patients. [4], [5] Studies in humans and in experimental animal models have demonstrated that after detachment of the retina, the photoreceptors begin to degenerate and die over time. [6]–[8] Therefore, therapeutic agents targeting photoreceptor death may improve treatment for retinal disorders associated with retinal detachment.

TUDCA is a minor component of human bile and a primary constituent of bear bile. [9], [10] Bear bile has been used in Chinese medicine for ophthalmic and hepatic indications for over 3000 years. [9], [10] Recently, researchers have tested TUDCA and related bile acids for biological activity using modern scientific methods. The related drug, ursodeoxycholic acid (UDCA) -also known as Actigall, Urso, or Ursodiol- reduces liver damage in the setting of cholestasis and has been approved by FDA for the treatment of primary biliary cirrhosis. TUDCA itself has been demonstrated to show cytoprotective effects in a variety of experimental systems including several models of neurodegenerative diseases [11]–[17] as well as against light-induced or oxidative stress-induced retinal damage in mice and mouse model of retinitis pigmentosa. [9], [18], [19]

This study tests whether the systemic administration of tauroursodeoxycholic acid (TUDCA) can protect photoreceptors from cell death after experimental retinal detachment. We show that this agent has neuroprotective effects, associated with inhibition of apoptosis and decrease in oxidative stress, thereby exhibiting potential as a novel neuroprotective therapeutic drug in eye diseases characterized by photoreceptor cell loss due to retinal detachment.

## Results

### TUDCA prevents photoreceptor death after retinal detachment

**Figure 1:**
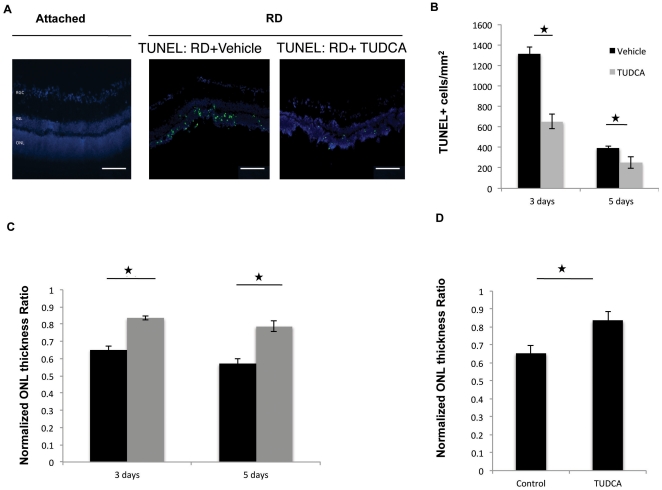
Systemic TUDCA administraton decreases photoreceptor cell loss in RD. (**A**) DAPI (blue) and TUNEL (green) staining of attached or detached for 5 days retina sections. TUDCA treatment was started 24 hrs before RD induction and continued daily at a dose of 500 mg/kg/day. (**B**) Quantitative analysis of TUNEL positive cells 3 and 5 days after RD with or without daily TUDCA treatment (500 mg/kg/day, I.P.). (n = 6, p<0.05). (**C**) Quantitative data exhibiting the protective effect of TUDCA (500 mg/kg/day, I.P.) on ONL thickness preservation, 3 and 5 after RD. (n = 6, p<0.05). (**D)** Protective effects of TUDCA were seen even when TUDCA treatment was started 6 hours after RD induction. (n = 6, P<0.05).

First, we assessed photoreceptor death after retinal detachment by TUNEL staining, which detects DNA fragmentation in apoptotic or necrotic nuclei. [20] Intraperitoneal administration of TUDCA (500 mg/Kg/day) significantly reduced the numbers of TUNEL-positive cells in outer nuclear layer (ONL) three days (651±68 mm^2^ vs 1314±68 mm^2^ in control group, P = 0.001) and five days (243.4±23.9 mm^2^ vs 393.7±14.4 mm^2^ in control group, P = 0.04) after retinal detachment (Fig. 1B).

Then we investigated the ability of systemically administered TUDCA to preserve ONL thickness after retinal detachment (Fig. 1C and 1D). The standardized ratio of ONL to total thickness in detached versus attached areas was measured and compared between vehicle and TUDCA treated groups. A ratio of 1 represents no loss of ONL thickness, while ratios less 1 than represent loss of ONL thickness. After three days of detachment, the ONL thickness ratio of the vehicle-treated group decreased to 0.65±0.03, while TUDCA significantly prevented the reduction of ONL thickness ratio (0.84±0.03, P = 0.0016, Fig 1C). Five days after detachment, the ratio in the vehicle treated groups was 0.57±0.04 whereas in the TUDCA treated group it remained higher at 0.79±0.03 (P = 0.002). Administration of TUDCA six hours after the induction of RD also showed efficient neuroprotection (P = 0.03; Fig. 1D).

### TUDCA does not affect TNF-α, MCP-1 or CD68+ cell infiltration

**Figure 2:**
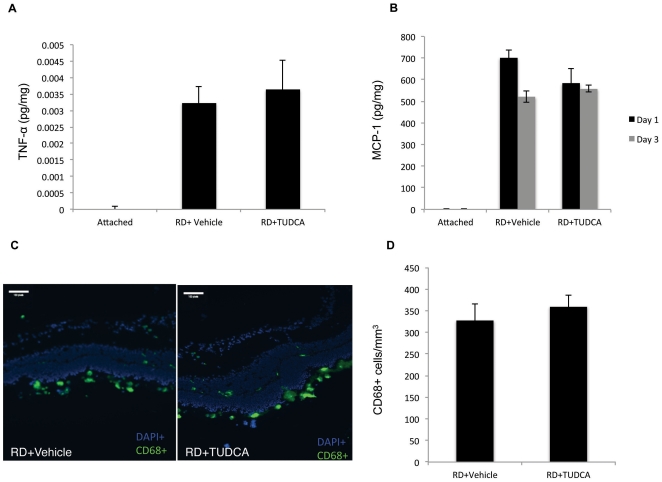
TUDCA did not diminish the inflammatory response after RD. ELISA for TNF-α (**A**) and MCP-1 (**B**) levels 1 day after RD. TUDCA was administered as in Figure 1. Immunostaining (**C**) and quantification (**D**) for infiltrating CD68+ cells (green) 1 day after RD. Nuclei were counterstained with DAPI (blue) TUDCA was administered as in Figure 1 (A) (n = 6, P>0.05).

We have shown previously that inflammatory cytokines and macrophage infiltration are markedly elevated in experimental RD. [21]–[23] Similar to those studies, increased levels of TNF-α, MCP-1 and CD68+ cell infiltration were noted after retinal detachment but were not altered by TUDCA treatment (Fig. 2).

### TUDCA reduces protein oxidization by reactive oxygen species (ROS)

**Figure 3:**
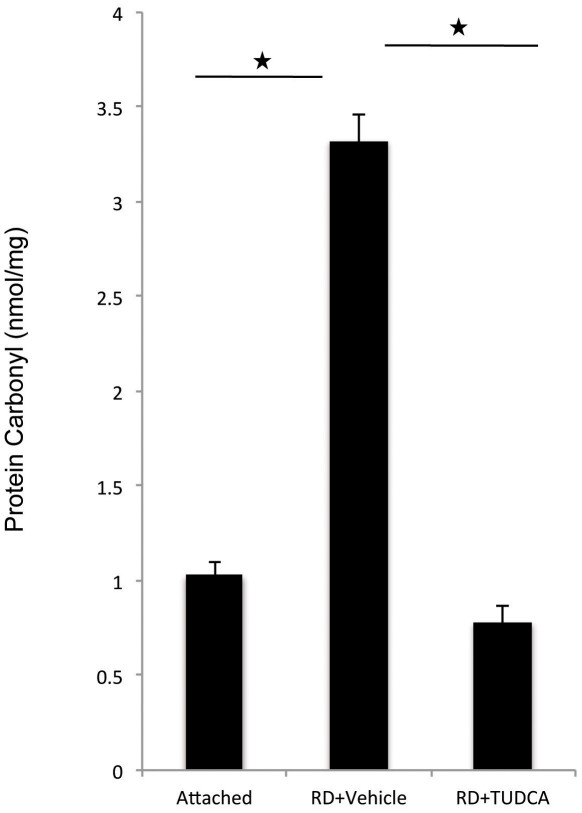
TUDCA diminishes oxidative stress after RD. Levels of protein carbonyl adducts can be used as an index of exposure to oxidative stress. The increase in the protein carbonyl concentration 24 hours after RD as measured by ELISA, was completely reversed with TUDCA treatment (administered as in Figure 1 (A), n = 6, p<0.05).

TUDCA has been proven to exert cytoprotective effects in different models by reducing oxidative stress. [19], [24] We have also previously shown that retinal detachment leads to increased ROS products such as increased levels of protein carbonyl content. [23], [25] One day after retinal detachment, TUDCA treatment completely prevented the increase in the amount of protein carbonyls after retinal detachment (Fig. 3).

### The neuroprotective effects of TUDCA are associated with decreased caspase activation

**Figure 4:**
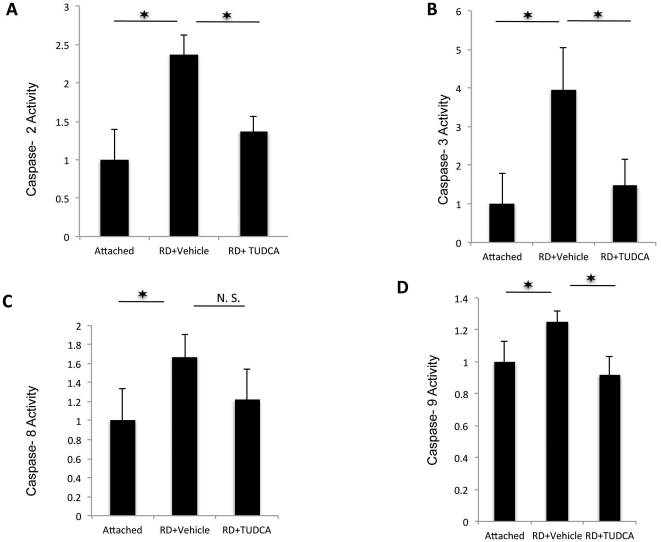
TUDCA's effect on various caspases after RD. Assay of Caspases 2, 3, 8, and 9 activity after RD and TUDCA treatment. Treatment of RD with TUDCA decreased the activation of caspases 2, 3 and 9 (n = 6, p<0.05) but not of 8 (n = 6, p>0.05).

**Figure 5:**
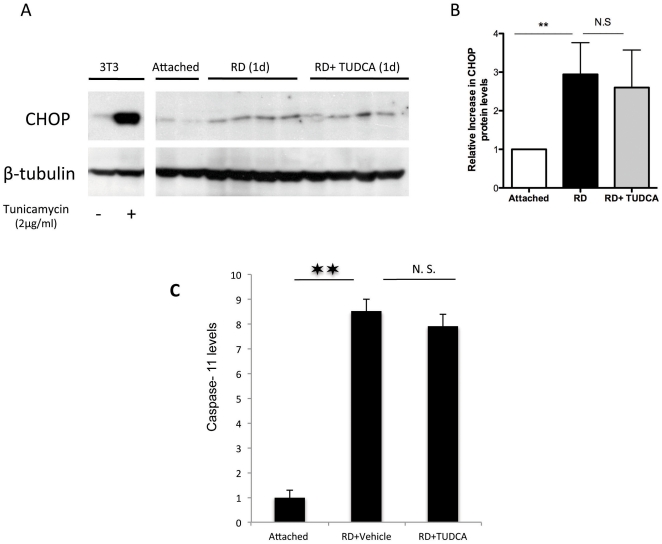
TUDCA treatment does not affect endoplasmic reticulum stress and Caspase 11 levels induced by RD. Western blots (**A**) and quantification (**B**) of CHOP expression in retinal lysates from attached and detached retinas treated with TUDCA or vehicle (administered as in figure 1). (n = 4 p<0.05 for RD vs attached). 3T3 fibroblasts and Tunicamycin (2microgram/ml for 6 hours) were used as a positive control. (**C**) Quantification of Caspase 11 levels after RD and TUDCA treatment (n = 6, p>0.05).

Caspases have previously been shown to play a role in retinal detachment. [26], [27] The cytoprotective effects of TUDCA were also investigated by measuring activity of caspases 2, 3, 8, 9 and 11, which includes pathway members from both the intrinsic and the extrinsic pathway. TUDCA administration significantly decreased retinal detachment-induced activity for Caspases 2, 3 and 9 (P<0.05), while it did not alter the increases in Caspase 8 and 11 (Fig. 4 and 5).

### TUDCA does not significantly decrease Endoplasmic reticulum (ER) stress after retinal detachment

Prolonged ER stress can lead to cell death. Recently Liu *et al.* [35] have shown that proapoptotic ER stress proteins such as growth arrest DNA damage-inducible gene 153 (GADD153) also known as C/EBP homologous protein [28], is increased after RD in rats. Additionally, in isolated rat pancreatic acini TUDCA has been shown to decrease ER stress and downregulate CHOP expression [36]. We thus evaluated the effect of TUDCA on CHOP expression after RD. Even though RD increased the levels of CHOP, systemic TUDCA administration did not have a significant effect on this elevation (Figure 5).

## Discussion

Hydrophilic bile acids like UDCA and TUDCA had been empirically used for ophthalmic indications in traditional Chinese medicine for thousands of years. [9], [10] Recently they have been studied more systematically and have been shown to be cytoprotective in experimental models of neurodegenerative diseases, including Huntington's disease [11], [12], Alzheimer's disease [13], Parkinson's disease [14] and hemorrhagic stroke. [15], [16] In mice, TUDCA also protects photoreceptors against light-induced retinal damage and from a genetic mutation that models retinitis pigmentosa. [9], [18], [19]

In this study, we demonstrate that systemic administration of the hydrophilic bile acid TUDCA has photoreceptor neuroprotective effects in an experimental retinal detachment model and in agreement with studies by others, where TUDCA has been shown to mediate its cytoprotective effects partially through inhibition of caspase mediated apoptosis. [29]–[31] The neuroprotective effect of TUDCA treatment in our model of experimental retinal detachment was correlated with inhibition of caspases 2, 3 and 9 and a decrease in TUNEL- positive cells. Daily TUDCA administration reduced the number of TUNEL positive cells by about 50% and reduced the loss of ONL thickness by a similar amount initially but the effect becomes less pronounced on day 5. This could be the result of the existence of alternative mechanisms of cell loss in retinal detachment as was recently described by our group. [28] It has been shown by others and us that TUNEL staining is not restricted to apoptotic cells but encompasses necrotic cells as well. [20], [28] It is possible that TUDCA is not effective in blocking both apoptotic and necrotic pathways that are activated upon RD and thus the protection offered by it may be limited.

Inflammation is thought to play a significant role in retinal detachment mediated photoreceptor cell loss. Several studies have shown upregulation of inflammatory cytokines such as TNF-α and MCP1 [32], [33] and have demonstrated increased infiltration of macrophages. [34] However, we showed that TUDCA did not affect the inflammatory cytokine production measured in total retina homogenates (it could affect the leukocyte MCP content) and did not alter the inflammatory cell infiltration after retinal detachment. Consistent with our findings, a study on hepatocytes showed that TUDCA did not affect the levels of the TNF-α released from Kuppfer cells isolated from the liver [32] while another study found that TUDCA protected hepatocytes from TNF-a induced death. [35] In a study of isolated biliary epithelial cells TUDCA did not alter the levels of IL-6 or MCP1 secretion. [36] These data suggest that the cytoprotective action of TUDCA may be mediated through a direct effect on damaged cells rather than through regulation of inflammatory processes.

Endoplasmic Reticulum (ER) stress has been shown to be a feature in various neurodegenerative disorders, [37] as well as in retinal detachment. [38] Persistent ER stress leads to pro-apoptotic molecule induction such as growth arrest DNA damage-inducible gene 153 (GADD153) also known as C/EBP homologous protein [28]. In a study of isolated pancreatic acini it was shown that TUDCA decreased ER stress and CHOP expression. [36] Similar to the previous study of ER stress in RD [35] we found that RD lead to increased levels of CHOP (Fig. 5) but in contrast to the pancreatic acini study TUDCA did not significantly alter its expression. In line with this finding, TUDCA did not decrease Caspase 11 induction, a downstream effector of CHOP.

The related drug UDCA has been shown to inhibit changes in mitochondrial transmembrane potential and ROS generation in isolated mitochondria from the liver of adult rats. [39] Oxidative stress is a factor playing a critical role in photoreceptor death after RD and we have shown that ROS reduction is associated with neuroprotective effect on photoreceptors after RD. [15], [23], [25] Administration of TUDCA led to almost complete abolishment of the increase in protein carbonyl content, a measure of ROS levels. Hence, a combination of the inhibition of caspases and decrease in the ROS levels could be partially responsible for the neuroprotective mechanism of TUDCA in the RD model.

The exact mechanism TUDCA protects photoreceptor cells remains unknown and its effects may be direct or indirect. Given the limited effect on inflammatory cytokines and infiltrating leukocytes, it seems that the inflammatory cell may not be a major target of TUDCA -at least not in this model. Thus, it seems that a direct effect on photoreceptor cells is more likely. This may also partially explain why the delayed administration of TUDCA lead to reduced efficiency since bioavailability of TUDCA to the outer retina is expected to be impaired after photoreceptor separation from the tissues supplying them with nutrients (RPE and choroid).

Although a previous study reported that systemic administration of TUDCA (500 mg/kg/day) in a model of retinitis pigmentosa had negative impact on the weight gain of developing mice, [18] there was no effect on the weight of adult rats in our study. This could be because our animals were already adults when treatment was started. In fact, the similar non-taurine conjugated UDCA does not show any significant adverse effects given to humans for extended period of time- up to 2 years- to treat liver diseases, and has been approved by the FDA. These findings suggest that TUDCA is an efficient and safe neuroprotective agent and may have a therapeutic potential for patients suffering from diseases where retinal degeneration is involved.

Photoreceptor loss and subsequent visual decline occurs when the photoreceptors are separated from the underlying retinal pigment epithelium. Physical separation of photoreceptors is seen in various retinal disorders, including retinal detachement as well as age-related macular degeneration [4] and diabetic retinopathy [5]. The visual acuity of patients with rhegmatogenous retinal detachment is not always restored, even after successful operation for reattachment. Two fifths of patients with rhegmatogenous retinal detachment involving the macula, a region essential for central vision recover 20/40 or better vision because of photoreceptor death. [40], [41] Thus, identification of neuroprotective agents preventing photoreceptor loss may open a novel approach for treatment of these diseases. The findings of this study suggest that systemic administration of TUDCA may be such an approach for preventing vision deficit in various retinal disorders associated with photoreceptor loss.

## Methods

### Animals

All animal experiments adhered to the ARVO Statement for the Use of Animals in Ophthalmic and Vision Research, and the protocols were approved by the Animal Care Committee of the Massachusetts Eye and Ear Infirmary. Adult male Brown Norway rats (Charles River, Wilmington, MA) weighing 200–250 g were fed standard laboratory chow and allowed free access to water in an air-conditioned room with a 12 h light/12 h dark cycle.

### Retinal Detachment Induction

Rats were anesthetized with 100 ml of a mixture of 5 parts Ketamine (100 mg/ml, Phoenix Pharmaceutical, Inc., St Joseph, MO), 4 parts PBS and 1 part Xylazine (100 mg/ml, Lloyd Laboratories, Inc., Shenandoah, IA). Body temperature was monitored and the animals were kept on a heating pad during the procedure as well as during the recovery period. The pupils were dilated with drops of phenylephrine 5%/tropicamide 0.5%. A partial retinal detachment was induced with a transscleral, subretinal injection of ∼50 mml of 1% sodium hyaluronate (Provisc, Alcon Laboratories Inc., Fort Worth, TX), as described previously.[8] The partial retinal detachments occupied about 40–60% of the fundus as seen under the operating microscope. Eyes that developed choroidal hemorrhage or significant vitreous hemorrhage were excluded prior to analysis.

### Treatment with TUDCA

Animals were treated daily with an intraperitoneal injection of vehicle (0.15M NaHCO_3_) or vehicle containing TUDCA (EMD Chemicals, Gibbstown, NJ) at a dose that has been shown safe and effective in previous studies, 500 mg/kg body weight. [11] The pH was adjusted to 7.4, which was allowed to dissolve at room temperature with vortexing as needed. With the exception of Figure 1D, all the animals received the first injection 24 hours prior to the induction of RD. For Figure 1D, the treatment was started 6 hours after RD. The animals of this group also received a vehicle injection 24 hours before the RD.

### ONL thickness ratio and TUNEL stain

The rats were euthanized 3 and 5 days after retinal detachment induction and the eyes were enucleated and fixed in paraformaldehyde 4% in phosphate buffer saline (PFA-PBS) at 4°C overnight. Then they were embedded in Optimal Cutting Temperature media (OCT- Tissue Tek; Sakura Finetec, Torrance, CA), frozen at −21°C and cut in 10mm- thick sections. These sections were then fixed in PFA 4% for 15 minutes, prior to staining with DAPI nuclear stain (AnaSpec/Eurogentec Group) and Terminal dUTP Nick-End Labeling (TUNEL) staining according to manufacturer's instructions (Apop Tag Fluorescein Apoptosis detection kit, Millipore, Billerica MA). Fluorescence was imaged (DM RXA miroscope; Leica, Solms Germany) and digitally captured. Using ImageJ image analysis software (http://rsbweb.nih.gov/ij/), the thickness of the outer nuclear layer (ONL) was measured in the detached as well as the attached retina. The thicknesses were measured based on the outer boundaries of the DAPI nuclear stain. Because the thickness of the retina seen in cross-section varies depending on the angle of the sectioning plane with respect to the retina, the outer nuclear layer thickness was normalized to the thickness of the entire retina. The “normalized ONL thickness ratio” is defined as the (ONL thickness/total thickness in detached retina)/(ONL thickness/total thickness in attached retina). Sections just posterior to the lens were used for thickness measurements. Next, the number of TUNEL- positive cells per mm^2^ was counted in a masked fashion at the central area of the detached retina and compared between the different treatment groups. All measurements were performed in 12 points of each section in 6 eyes per group.

### Immunohistochemistry

In order to assess macrophage infiltration in detached retinas (N = 6 eyes per group), the sections were fixed in PFA-PBS 4% overnight, blocked in skim milk, and incubated with anti-CD68 monoclonal antibody (Millipore, clone ED-1, #MAB1435) overnight. After washing with PBS, the sections were incubated with the secondary antibody Alexa Flour 488-conjugated goat anti-mouse IgG (Invitrogen, Carlsbad,CA) for 2 hours**,** washed again and mounted in Permafluor (Thermo Scientific, Fremont, CA). The number of CD68+ cells per mm^3^ was determined using the same methods as described above for TUNEL-positive cell quantification.

### Enzyme Linked Immunosorbent Assays

Eight eyes from each group (retina attached, and retina detached with or without treatment with TUDCA) were analyzed. Each retina was dissected and lysed in 200 ml of protein lysis buffer [42], sonicated for 10 seconds and then centrifuged for 5 min at 10,000 rpm, 4°C. The assays were performed on the supernatants. For protein carbonyl content (Cell Biolabs, San Diego, CA) and TNF-a (R&D Systems Inc., Minneapolis, MN) assays, the samples were collected 24 hours after RD, while for MCP-1 assays (Thermo Scientific, Rockford IL) samples were collected both at 24 and 72 hours. To measure the concentration of rat MCP-1, TNF-α and protein carbonyl, the retina was sonicated in lysis buffer and equal protein extracts were used in ELISA assays. The assays were performed according to manufacturer's instructions and the results were normalized for the protein concentration in the sample using a Bio-Rad Protein Assay Kit (Bio-Rad Laboratories, Hercules, CA #500-0002).

### Caspase 2, 3, 8, 9 Colorimetric Assay

The caspase assays were performed on the retina of animals without retinal detahcment (n = 6) as well as animals with retinal detachment that received vehicle (n = 6) or TUDCA (n = 6). The samples were stored in lysis buffer, sonicated for 10 seconds, and centrifuged at 10,000 rpm for 5 minutes. The assay was performed on the supernatant according to manufacturer's instructions (Casp-3-C and Casp-8-C for caspases 3 and 8, Sigma-Aldrich, Saint Louis, MO; APT163 and APT173 for caspases 2 and 9, Chemicon-Millipore, Temecula,CA) This assay is based on the detection of a chromophore (p- nitroalanine) which is released upon enzymatic cleavage of the labeled substrate DEVD-*p*NA from the activated caspases of the cells undergoing apoptosis. The optical density was assessed at 405nm and normalized for protein concentration, initial optical density, and compared to a sample filled with buffer.

### Western Blotting

The vitreous and neural retina, combined, was collected on day 3 after retinal detachment. Samples were run on 4% to 12% SDS-polyacrylamide gel electrophoresis and transferred onto PVDF membrane. After blocking with 3% nonfat dried milk, the membrane was reacted with a CHOP (1∶1000 ; Cell Signaling, Cat #5554S), caspase-11 (1∶1000 ; Santa Cruz Biotechnology, Cat #SC-28230). Ôhey were then developed with enhanced chemiluminescence. â-tubulin (1∶1,000; Cell Signaling Technology, Cat #2146) was used as a loading control.
